# A Community Effort: Combining Functional Amplicon Sequencing and Metagenomics Reveals Potential Biosynthetic Gene Clusters Associated with Protective Phenotypes in Rhizosphere Microbiomes

**DOI:** 10.1128/mSystems.00587-21

**Published:** 2021-06-08

**Authors:** Jaclyn M. Winter

**Affiliations:** a Department of Medicinal Chemistry, University of Utah, Salt Lake City, Utah, USA

**Keywords:** bioinformatics, dom2BGC annotation pipeline, soil microbiome, biosynthetic gene cluster

## Abstract

Soil-dwelling microorganisms associated with plant roots carry out essential processes that promote plant growth and productivity. In addition to these beneficial functions, the rhizosphere microbiome also serves as the first line of defense against many plant pathogens. While many rhizobacteria are capable of producing antifungal natural products, fungal pathogens, such as those belonging to the genus Fusarium, continue to be a major threat to agricultural crops worldwide. In this issue, Tracanna and coworkers (V. Tracanna, A. Ossowicki, M. L. C. Petrus, S. Overduin, et al., mSystems 6:e01116-20, 2021, https://doi.org/10.1128/mSystems.01116-20) implement a targeted amplicon sequencing approach to identify conserved domains and specific metabolic pathways shared among soil samples with antagonistic activities against Fusarium culmorum. They also introduce dom2BGC, an open-source annotation platform that builds co-occurrence networks of natural product-associated domains across samples and aids in putative gene cluster reconstruction. When coupled with metagenomics, functional amplicon sequencing and the dom2BGC pipeline can aid in identifying mechanisms and potential metabolites associated with particular microbiome-associated phenotypes.

## COMMENTARY

Soils are biologically diverse habitats and represent some of the most complex ecosystems found in nature (1). As sustainable agricultural practices are dependent on soil quality and health, research efforts have focused on elucidating the community composition of the soil biota and their functional attributes. Metagenomic approaches have been instrumental in this endeavor and have aided in the identification of dominant phylotypes and essential ecosystem functions such as decomposition of organic matter, soil detoxification, and nutrient recycling (2, 3). Because of their profound effects on plant growth and health, a number of studies have also focused on deciphering the microbiome associated with plant roots and characterizing their mutualistic relationships (4, 5).

Investigations into the rhizosphere have shown that this is a complex and dynamic environment (6). Plants use root exudates to help recruit and interact with the rhizosphere microbiome (7), and the rhizobacteria perform specific biogeochemical processes for the host, including protection against pathogenic bacteria and fungi. Because of this host resistance phenotype, the organisms that are responsible for eliciting the protective activity are gaining traction as resources for the discovery and development of new anti-infective agents. Unfortunately, due to sequencing limitations in capturing the full genomic diversity within a sample coupled with deficiencies in soil macroecological research (8), many of the bioactive organisms remain hidden in the rhizosphere microbiome. Additionally, as a majority of the microorganisms responsible for the protective phenotype cannot yet be cultivated in a laboratory setting, culture-independent approaches must be implemented to identify the associated mechanisms of protection. Recent success using a metagenomics-based screening technique with diverse soil samples led to the discovery of the malacidins, cyclic lipopeptides with antibiotic activity against multidrug-resistant Gram-positive bacterial pathogens (9).

In addition to pathogenic bacteria, soilborne fungal plant pathogens are a serious threat to crops worldwide, with Fusarium species being among the most devastating. While a previous study identified soil samples with antagonistic activity against Fusarium culmorum (10), the causative agent of root rot and head blights in wheat and barley, taxonomic comparisons of the rhizobacteria failed to reveal any taxa that were unequivocally responsible for the observed antifungal activity. As the protective phenotype could be attributed to specialized metabolites produced by low-abundant bacteria in the rhizosphere microbiome or by pathways that are unevenly distributed across taxa due to horizontal gene transfer, Tracanna et al. (11) adopted a targeted sequencing-based approach to elucidate the metabolic pathways associated with the antifungal phenotypes.

To identify putative antifungal-producing clusters in disease-suppressive rhizobacteria, the authors focused on biosynthetic machinery responsible for making nonribosomal peptides. Nonribosomal peptides (e.g., lipopeptides and siderophores) are a large class of bioactive natural products and are typically biosynthesized in an assembly line-like fashion by nonribosomal peptide synthetases (NRPSs). Adenylation domains are essential components of the NRPS assembly line and are responsible for selecting the amino acid building blocks that get incorporated into the natural product. Using degenerate primers, adenylation domains were PCR amplified from rhizosphere DNA collected from four of the authors’ soil samples possessing activity against *F. culmorum* and four that did not. One of the samples with strong activity against *F. culmorum* was also used for high-quality metagenomic sequencing. From the PCR-generated adenylation domains, the authors found that the diversity of unique amplicons obtained from the soil biome samples was equal to the level of diversity found across all publicly available genomes. This finding further highlights the untapped potential of soil-dwelling microorganisms for natural product discovery programs (12). Furthermore, it was reported that the diversity of adenylation domains obtained from the functional amplicon approach was much higher than what was identified in the metagenomic data set. These data demonstrate that targeted PCR-based approaches can bring to light biosynthetic gene clusters from low-abundant genomes which are otherwise inaccessible using traditional metagenomic sequencing.

With the adenylation sequences from the rhizosphere microbiomes in hand, the authors developed an automated annotation platform to assist with comparative analyses across the sample sets. Briefly, dom2BGC annotates biosynthetic domains based on similarities with *in silico* amplicons extracted from the antiSMASH and MIBiG databases. Taxonomic diversity and community structure relationships are calculated across sample sets, and domain co-occurrence networks establish biosynthetic distribution patterns that can be used to explain microbiome-associated phenotypes. While a similar PCR-based targeting approach was used in the discovery of the malacidins, the bioinformatics program eSNaPD (13) that initially identified the biosynthetic cluster from global microbiome samples is currently not accessible, emphasizing the importance of maintaining open source platforms. The source code for dom2BGC is available through GitHub.

Using the dom2BGC platform, the authors identified community structure overlap between adenylation domain profiles from suppressive rhizobacteria and were able to map the sequences to multiple taxonomic groups. From the co-occurence patterns, dom2BGC was also successful at reconstructing multiple biosynthetic gene clusters. Results from the amplicon clustering were validated using 10× metagenome assembly and demonstrated that dom2BGC is a powerful platform for identifying functional elements in complex microbial communities. Detailed analysis of the amplified adenylation domains suggested an enrichment of cyclic and branched lipopeptide-producing biosynthetic clusters in the soil samples possessing antifungal activity, and bioinformatic analysis of the metagenomic data revealed several siderophore, lipopeptide, and 2,4-diacetylphloroglucinol gene clusters. It was speculated that the agents associated with the respective gene clusters could be responsible for the antagonistic activity against *F. culmorum*.

Tracanna and coworkers (11) demonstrate that degenerate PCR-based screening can be used as a diagnostic tool for assessing the disease-suppressive potential of agricultural soils. dom2BGC, when combined with functional amplicon sequencing and metagenomics, can help uncover metabolic pathways responsible for microbiome-associated phenotypes and provide insight into potential protective agents. While this study was focused on interrogating the rhizosphere microbiome, the techniques and platforms discussed can be applied to other complex biome samples such as the human microbiome. Having access to these tools will enable our ability to tap into the chemical diversity of microbiomes and aid in the discovery of new anti-infective agents.
